# Supplementary material for: Isolation of Cherry Seed Oil Using Conventional Techniques and Supercritical Fluid Extraction
Source: Foods. 2022 Dec 20;12(1):11. doi: 10.3390/foods12010011 (PMC9818375; doi:10.3390/foods12010011)
Supplement: Supplementary file 1 [file foods-12-00011-s001.zip › foods-2036400-supplementary.pdf]

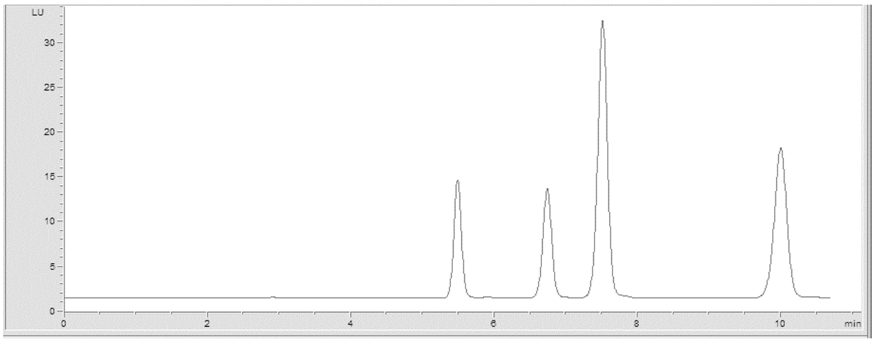

Figure S1. Chromatogram of tocopherol standards (α-, β-, γ-, δ-tocopherol, respectively)

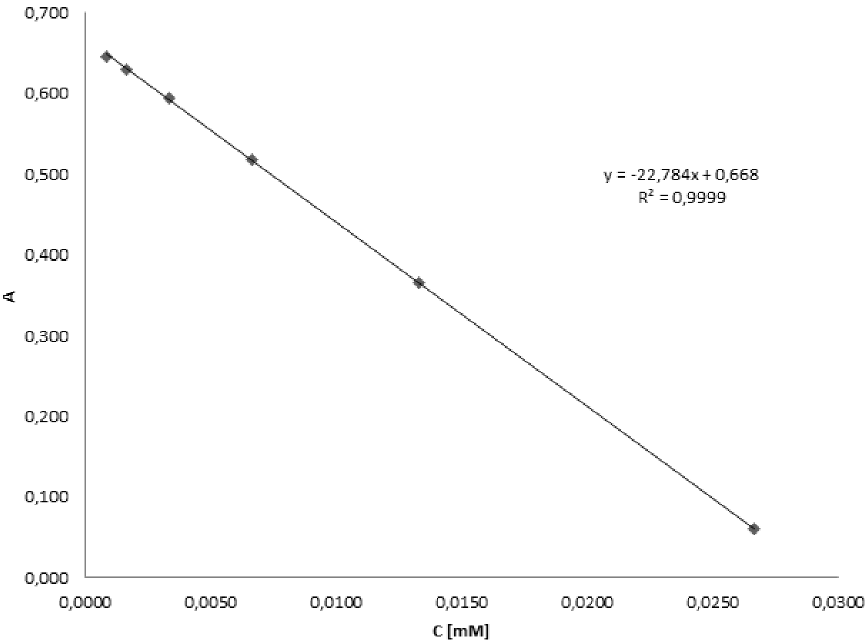

Figure S2. Calibration curve and the equation for DDPH assay

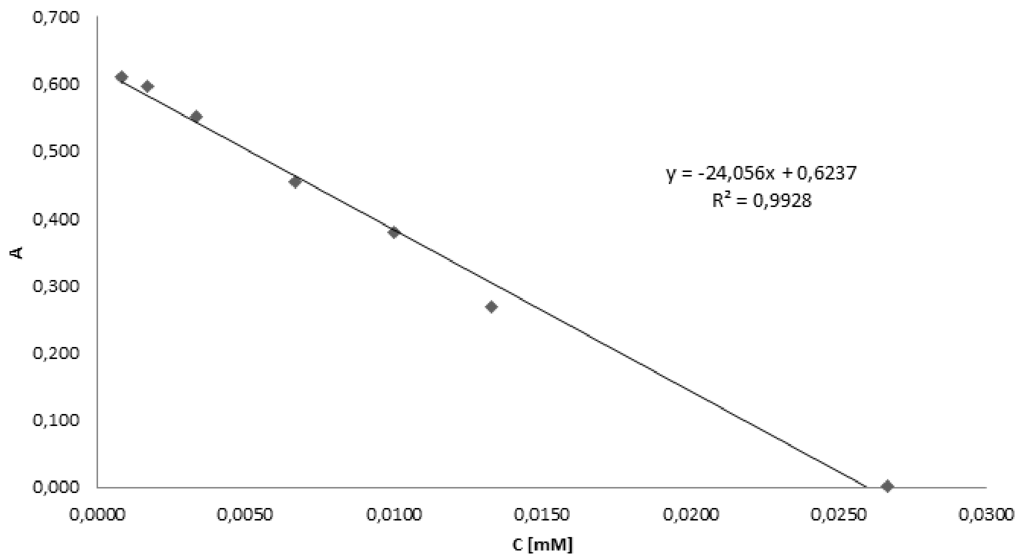

Figure S3. Calibration curve and the equation for ABTS assay

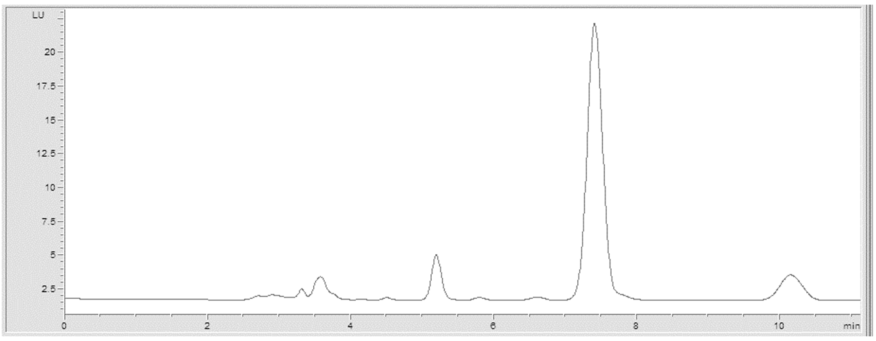

Figure S4. Chromatogram for tocopherol analysis of SFE-1 experiment (350 bar, 70 °C, 0.3 kg CO_2/h)
